# One-Dimensional-Like Titania/4′-Pentyl-4-Biphenylcarbonitrile Composite Synthesized Under Magnetic Field and its Structure–Photocatalytic Activity Relationship

**DOI:** 10.3389/fchem.2018.00370

**Published:** 2018-09-11

**Authors:** Nur I. Abu Bakar, Sheela Chandren, Nursyafreena Attan, Wai L. Leaw, Hadi Nur

**Affiliations:** ^1^Centre for Sustainable Nanomaterials, Ibnu Sina Institute for Scientific and Industrial Research, Universiti Teknologi Malaysia, Johor, Malaysia; ^2^Department of Chemistry, Faculty of Science, Universiti Teknologi Malaysia, Johor, Malaysia; ^3^Centre Laboratory of Minerals and Advanced Materials, Faculty of Mathematics and Natural Science, Universitas Negeri Malang (State University of Malang), Malang, Indonesia

**Keywords:** one-dimensional-like titania composite, liquid crystal, magnetic field, electron mobility, photocatalytic activity

## Abstract

The demonstration of the structure–properties relationship of shape-dependent photocatalysts remains a challenge today. Herein, one-dimensional (1-D)-like titania (TiO_2_), as a model photocatalyst, has been synthesized under a strong magnetic field in the presence of a magnetically responsive liquid crystal as the structure-aligning agent to demonstrate the relationship between a well-aligned structure and its photocatalytic properties. The importance of the 1-D-like TiO_2_ and its relationship with the electronic structures that affect the electron–hole recombination and the photocatalytic activity need to be clarified. The synthesis of 1-D-like TiO_2_ with liquid crystal as the structure-aligning agent was carried out using the sol–gel method under a magnetic field (0.3 T). The mixture of liquid crystal, 4′-pentyl-4-biphenylcarbonitrile (5CB), tetra-*n*-butyl orthotitanate (TBOT), 2-propanol, and water, was subjected to slow hydrolysis under a magnetic field. The TiO_2_–5CB took a well-aligned whiskerlike shape when the reaction mixture was placed under the magnetic field, while irregularly shaped TiO_2_–5CB particles were formed when no magnetic field was applied. It shows that the strong interaction between 5CB and TBOT during the hydrolysis process under a magnetic field controls the shape of titania. The intensity of the emission peaks in the photoluminescence spectrum of 1-D-like TiO_2_–5CB was lowered compared with the TiO_2_–5CB synthesized without the magnetic field, suggesting the occurrence of electron transfer from 5CB to the 1-D-like TiO_2_–5CB during ultraviolet irradiation. Apart from that, direct current electrical conductivity and Hall effect studies showed that the 1-D-like TiO_2_ composite enhanced electron mobility. Thus, the recombination of electrons and holes was delayed due to the increase in electron mobility; hence, the photocatalytic activity of the 1-D-like TiO_2_ composite in the oxidation of styrene in the presence of aqueous hydrogen peroxide under UV irradiation was enhanced. This suggests that the 1-D-like shape of TiO_2_ composite plays an important role in its photocatalytic activity.

## Introduction

Photocatalysis or photocatalytic reaction is defined as a chemical reaction that is accelerated by the photoabsorption of a solid material, or photocatalyst (Ohtani, 2008). A photocatalytic process begins with the absorption of light radiation, which has the equivalent or higher energy than the band gap energy of the photocatalyst particle. This creates photogeneration of electrons and holes in the valence band and conduction band. These electrons and holes can be used in the redox reaction, where the holes and electrons will react with the electron donor or acceptor species adsorbed on the particle surface (Linsebigler et al., 1995). The focus of this work is to utilize the most promising photocatalyst, which is titania (TiO_2_). TiO_2_ has been chosen as the metal oxide in this study because it is currently the most important, widespread, and highly studied metal oxide due to its low toxicity, high thermal stability, and broad applicability. With its semiconducting properties, TiO_2_ has shown outstanding performance in photocatalysis and self-cleaning applications (Fujishima et al., 2000).

To the best of our knowledge, there has been no rigid conclusion on the main factor that affects the photocatalytic activity, even though many studies have been carried out to modify the properties such as the surface area (Cheng et al., 2014); pore structure in terms of size, volume, and shape (Rasalingam et al., 2015); band gap energy (Lee et al., 2005); and crystalline phase (Tanaka et al., 1991; Ouzzine et al., 2014) of TiO_2_. The enhancement of the photocatalytic activity by adjusting these factors remains the focus in the research field of TiO_2_ photocatalysts (Nakata and Fujishima, 2012). In fact, the electron–hole recombination rate of the TiO_2_ photocatalyst could be the most prominent factor affecting photocatalytic activity. This is mainly because the irradiation of ultraviolet (UV) light onto the photocatalyst during the photocatalytic reaction process promotes the excitation of the electron from the valence band to the conduction band, leaving a hole in the valence band. These electrons and holes will be responsible for the chemical reactions, namely the reduction and oxidation processes. Therefore, a rapid recombination rate between the electrons and holes will reduce the photocatalytic activity.

Various efforts have been put together to decrease the rate of electron–hole recombination in TiO_2_ photocatalyst, i.e., the addition of noble metals (Papp et al., 1993; Rupa et al., 2009); doping with transition metal cations (Choi et al., 1994; Prasad et al., 2009), anions (Yu et al., 2002; Diwald et al., 2004; Ao et al., 2010), and metalloids (Xu et al., 2009); structural dimensionality (Xia et al., 2003; Feng et al., 2014); and formation of heterojunctions allow for the efficient charge separation (Wang et al., 2014). In the past few decades, the one-dimensional (1-D) structure of TiO_2_ has attracted more attention when compared with the two-dimensional and three-dimensional structures (He et al., 2015). It has been reported that the fiber, rod, wire, and tube-like materials were considered as the 1-D structure (Xia et al., 2003). The 1-D structure of materials has unique electronic properties, since it has been reported that the structure of 1-D materials can decrease the electron–hole recombinations (Xia et al., 2003). In comparison with the three-dimensional structure of materials, the probability of an electron to recombine with a hole is reduced ca. 33% since the 1-D structure has one degree of freedom compared with the three-dimensional structure, which has three degrees of freedom.

Various strategies have been undertaken for the preparation of the 1-D structure such as sol–gel template method, chemical vapor deposition, and hydrothermal method (Wu and Yu, 2004; Attar et al., 2009; Lia et al., 2009). It was also reported that titanium dioxide with controlled shapes was synthesized with the solvothermal technique, which saw the shape of TiO_2_ change from rhombic to truncate rhombic and to sphere. Synthesis of nanoparticles using a liquid crystal system has been reviewed (Hegmann et al., 2007). The liquid crystal system could be formed using a thermotropic liquid crystal or lyotropic liquid crystal. The first successful attempt of producing organized nanomaterial arrays with the aid of thermotropic liquid crystal was demonstrated by Patrick and coworkers (Mougous et al., 2000). Other examples of controlling the orientation of anisotropic nanomaterials using thermotropic nematic liquid crystals include the levitation of nickel nanowires in a twisted nematic cell (Lapointe et al., 2004, 2005) and also alignment of carbon nanotubes in low-molecular mass nematic liquid crystals (Dierking et al., 2004; Duran et al., 2005) and nematic elastomers (Courty et al., 2003). Needle-shaped, micron-sized semiconducting SiC particles were also successfully synthesized by Patrick and coworkers using nematic liquid crystal as the aligning agent. Organized arrays of high-aspect ratio nanotubes and nanofibers have also been synthesized using high molecular mass thermotropic liquid crystals (Chan et al., 2005).

Experimental findings proved what was theorized; that the 1-dimensional structure of the photocatalyst contributes to the making of a good photocatalyst. In this research, we propose a new method to synthesize 1-D-like photocatalyst via the sol–gel method under a magnetic field (0.3 T), with the aid of 4′-pentyl-4-biphenylcarbonitrile (5CB), which is a type of liquid crystal, as the structure-aligning agent. This was followed by physical characterizations using several spectroscopic methods with the aim to show that the 1-D-like structure effects the recombination rate of electron–hole as well as the photocatalytic performance.

## Experimental

### Materials

The materials used in this research were 4′-pentyl-4-biphenylcarbonitrile (5CB) (Sigma-Aldrich), which is a type of liquid crystal, tetra-*n*-butyl orthotitanate (TBOT) (Sigma-Aldrich) as the TiO_2_ precursor, 2-propanol, and distilled water.

### Synthesis of 1-D-like TiO_2_–5CB under magnetic field

In a typical experiment, 5CB (9.8 mg), 2-propanol (2.057 ml), and distilled water (0.016 ml) were mixed well in a 5-ml sample bottle. The TBOT (0.1 ml) was then added dropwise into the mixture, with stirring. Then, the solution was transferred into a petri dish and covered with a perforated aluminum foil. The petri dish containing TBOT, 5CB, 2-propanol, and distilled water was then placed under a magnetic field (0.3 T) produced by neodymium block magnet bars as shown in Supplementary Data 3. The sample was allowed to dry at room temperature until a constant weight was reached. The TiO_2_–5CB sample that was dried under the neodymium block magnet bars took 14 days to reach constant weight. The TiO_2_–5CB sample that was dried outside the neodymium block magnet bars took 12 days to reach constant weight. The magnetic strength applied was measured by a handheld Gauss Meter teslameter. The relative humidity was 60%.

### Samples characterization

The scanning electron microscopy (SEM) images of the TiO_2_ composites were taken using a JEOL JSM-6390LV instrument operating with an accelerating voltage of 15 kV. The specific surface area of the composites was obtained using a multipoint Brunauer–Emmett–Teller (BET) analysis via the measurement of nitrogen adsorption–desorption as a function of relative pressure. The samples were analyzed using a Thermo Fischer Scientific Sorptomatic 1990 surface analyzer utilizing a customary process at −196°C. For preparation, the samples were pretreated and degassed at 150°C for 12 h. Thermogravimetric analysis (TGA) was carried out using a Mettler Toledo TGA/SDTA851 thermogravimetric/differential thermal analyzer. The scanning was performed up to the temperature of 500°C, with a heating rate and air flow rate of 10°C min^−1^ and 20 mL min^−1^, respectively. Direct current (DC) electrical conductivity was tested in the range of 0 to 25 V at room temperature using a PHYWE Model with the main voltage of 230 V and frequency of 50/60 Hz. Hall effect studies were set up with the components of Hall probe (Ge Crystal–n-type), Hall probe wooden stand, Hall effect set up model (DHE)−21, electromagnet model (EMU)−50 V, constant power supply (DPS)−50, and digital gaussmeter model (DGM)−201. The current and Hall voltage values were recorded. Diffuse reflectance UV–visible (DR UV–Vis) spectroscopy was used to determine the chromophore–chromophore interactions in the samples. For the DR UV–Vis study, the spectra were recorded in the range of 200–500 nm using a Perkin Elmer UV–visible Spectrometer Lambda 900 with barium sulfate (BaSO_4_) as the standard reference. For Fourier-transform infrared spectroscopy (FTIR), the spectra were recorded in the range of 500–4,000 cm^−1^ using a Perkin Elmer Spectrum One Spectrometer. For X-ray photoelectron spectroscopy (XPS) analysis, the samples were analyzed using a PHI-5500 spectrometer coupled with monochromatic Mg K_α_ (1253.6 eV). The sample was scanned at a binding energy of 0 to 1200 eV with an X-ray source generated at 14 kV and 300 W. The crystallinity and phase content of the solid materials were determined using a Bruker AXS Advance D8 X-ray diffractometer (XRD) using a diffracted monochromatic beam of Cu K_α_ (λ = 1.5406 Å) radiation produced at 40 kV and 40 mA. The diffraction pattern was scanned in the 2θ range of 20–80° with a gradual increment of 0.05° and a step time of 1 s. Photoluminescence (PL) analysis was done to study the electrons–holes recombinations of the photocatalysts. The measurement was performed at room temperature using a JASCO spectrofluorometer (FP-8500), with a 150 W Xe lamp as the excitation source. The excitation wavelength was 279 nm.

### Photocatalytic testing

The photocatalytic activity of the TiO2 composites was evaluated through the photocatalytic oxidation of styrene in the presence of 30% aqueous hydrogen peroxide (H_2_O_2_). The reaction solution was prepared using 0.575 mL of styrene, 0.817 mL of aqueous H_2_O_2_, and 5 mL of acetonitrile. About 50 mg of photocatalyst was then added to the solution. The final concentration of styrene, hydrogen peroxide, and acetonitrile were 0.78, 1.25, and 15.02 M, respectively. The reaction was carried out for 24 h at room temperature with the solution being photoirradiated using a 2 × 15 W UV lamp (Vilber Lourmat, VL-215-C, 254 nm) and magnetically stirred continuously. The setup of experiment is attached as Supplementary Data 1. After the reaction, the mixture was centrifuged, and the catalyst was removed. The solution obtained was analyzed by a gas chromatograph equipped with a capillary column and a flame ionization detector. Nitrogen gas was used as the carrier gas with a column flow of 3 mL min^−1^.

## Results and discussion

### Formation of 1-D-like TiO_2_–5CB and its physical properties

**Figure 1:**
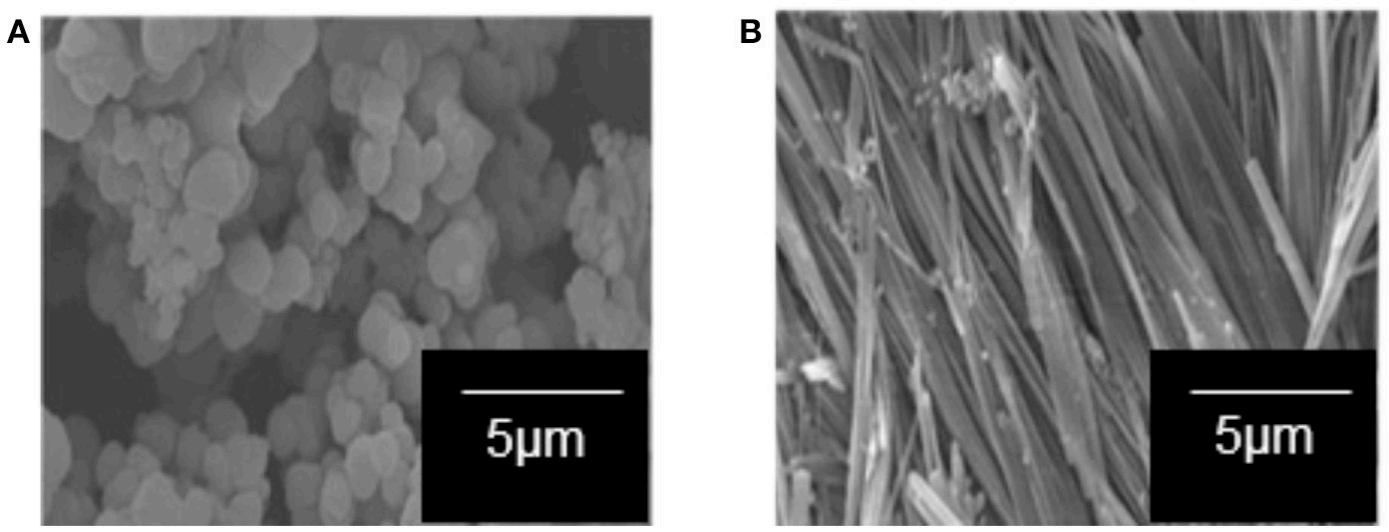
The SEM images of the TiO_2_ sample synthesized in the presence of 5CB liquid crystal **(A)** without magnetic field and **(B)** under magnetic field.

One-dimensional-like TiO_2_ composite was successfully prepared by the sol–gel method under a magnetic field (0.3 T) using TBOT as the TiO_2_ precursor, 5CB liquid crystal as the structure-aligning agent, and a mixture of 2-propanol and distilled water as the solvent. Figure 1A shows the SEM image of TiO_2_ composite synthesized without a magnetic field. The sample formed was not aligned and possesses irregular shape. Figure 1B shows the TiO_2_–5CB composite synthesized under a magnetic field. This composite was whiskerlike in shape. The 1-D-like TiO_2_–5CB synthesized under a magnetic field has a very high length-to-diameter ratio with a diameter and length in the range of 100 to 500 nm and 500 to 1,000 μm, respectively. The SEM images strongly confirm that this method can produce the 1-D-like TiO_2_ composites. In addition, the magnetic field does affect the shape of the TiO_2_ composite.

**Figure 2:**
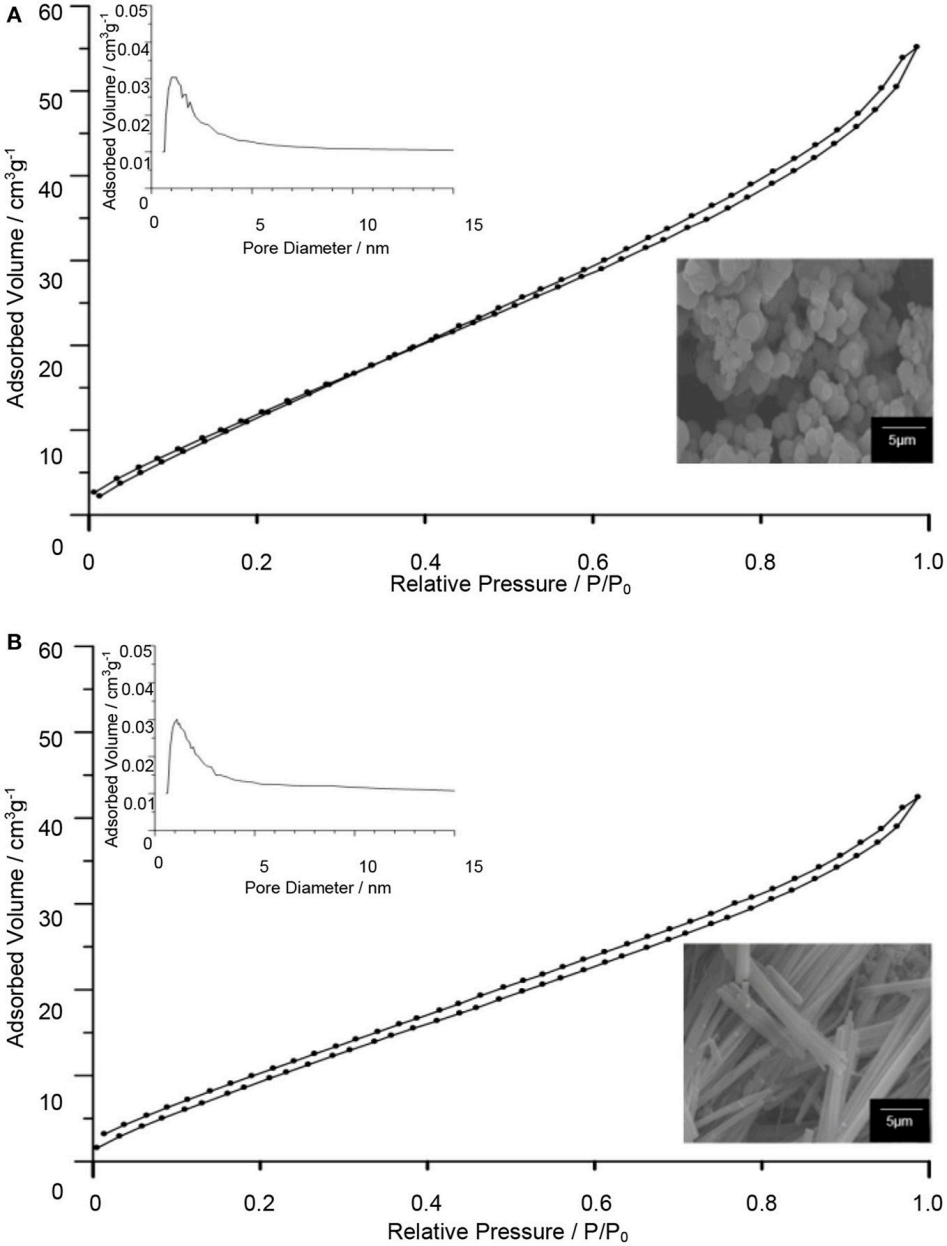
N_2_ adsorption–desorption isotherms and BJH plot for pore size distributions (inset) of TiO_2_–5CB synthesized **(A)** without magnetic field and **(B)** under magnetic field.

**Figure 3:**
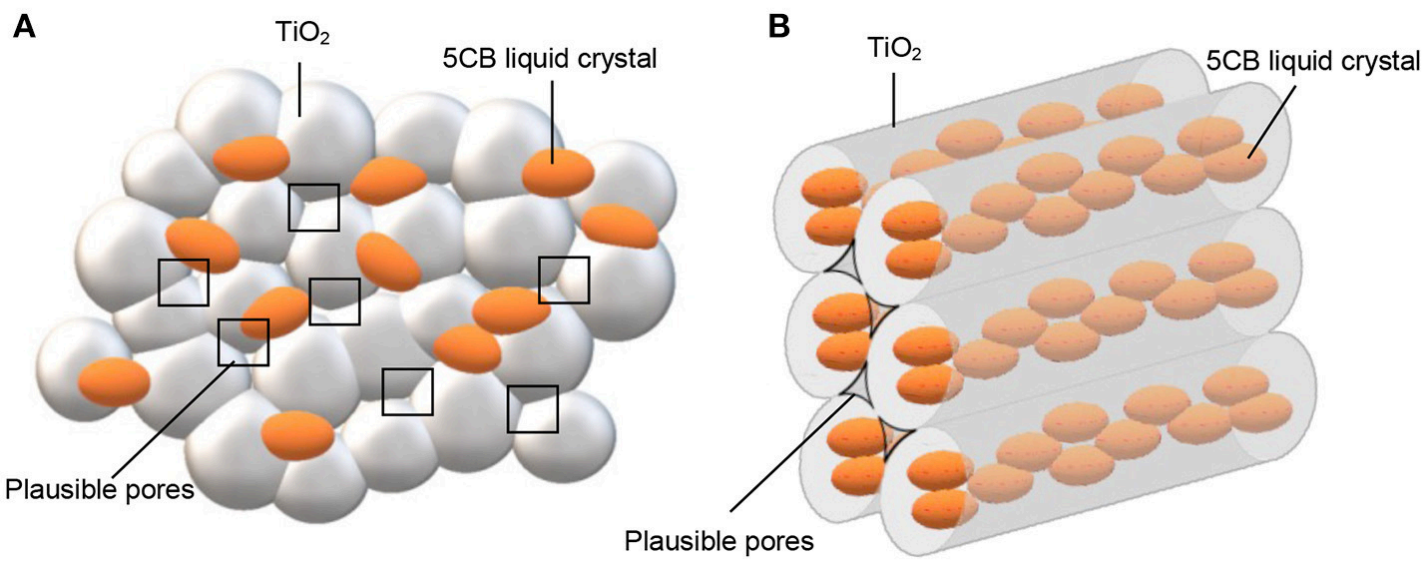
Plausible position of pores for TiO_2_ composites synthesized **(A)** without magnetic field and **(B)** under magnetic field.

The surface properties of TiO_2_–5CB composites were evaluated using multipoint the BET analysis. As shown in Figure 2, both types of TiO_2_ composites have similar isotherms, which is type IV isotherm with hysteresis loops of H4 (Ertl et al., 1997; Lakhi et al., 2017). The Barrett-Joyner-Halenda (BJH) plot analysis confirmed that TiO_2_–5CB synthesized under and without a magnetic field consisted of both micropore and mesopore with a uniform pore size distribution. The plausible location of pores in the TiO_2_ composites is illustrated in Figure 3. It was suggested that the pores were formed between the composites. From the sorption isotherm of the samples, the BET surface area was obtained and has been tabulated in Table 1. Both samples have similar surface areas. Physically, the surface area and pore structure of TiO_2_–5CB synthesized under and without the magnetic field are similar, and the only distinct difference is the shape of particles that could have a direct influence on the photocatalytic performance as discussed later.

**Table 1:** Surface area, pore volume, and pore size of TiO_2_ anatase standard (Sigma Aldrich, 637254), TiO_2_–5CB, and TiO_2_–5CB composites synthesized under and without magnetic field.

**Sample**	**Surface area (m^2^/g)**	**Pore volume (cc/g)**	**Pore size (nm)**
TiO_2_ anatase standard (Sigma Aldrich, 637254)	45	–	11.9
TiO_2_–5CB composites synthesized under magnetic field	42	0.08	1.03
TiO_2_–5CB composites synthesized without magnetic field	34	0.10	1.09

**Figure 4:**
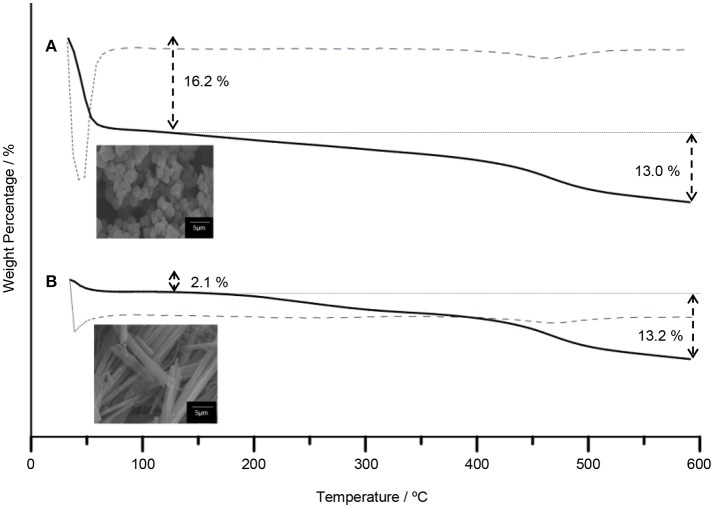
The TGA curves of TiO_2_ synthesized **(A)** without magnetic field and **(B)** under magnetic field.

The TiO_2_ composites were analyzed using the TGA. The TGA of TiO_2_ anatase standard is attached as Supplementary Data 2. Generally, the weight percentages of composites decreased with increasing temperature until 550°C as shown in Figure 4. The decomposition of 5CB occurred in the range of 130–550°C. The composition of 5CB in the TiO_2_–5CB prepared under and without a magnetic field is about 13.2 and 13.0 wt%, respectively. The amount of 5CB obtained from TG analysis was compared with the amount of 5CB added during the synthesis of TiO_2_–5CB. As shown in Table 2, it was confirmed that the content of 5CB in both samples did not vary significantly. The weight lost detected below 130°C corresponds to the evaporation of the solvent in the composites. It was found that the TiO_2_–5CB synthesized without the magnetic field contained a higher amount of solvent. One-dimensional-like TiO_2_–5CB synthesized under the magnetic field consisted of only 2.1% of solvent, which is 14.1% lower. This phenomenon explains the differences in the particle packing of each composite. The TiO_2_–5CB composite synthesized without the magnetic field is believed to consist of a large number of voids, which are formed by the loosely packed irregular-shaped particles. The voids formed are able to trap a higher amount of solvent. On the contrary, 1-D-like TiO_2_–5CB composites synthesized under a magnetic field had a more compact particle packing since the particles were uniform in shape. These particles were arranged tightly to each other, leaving negligible voids that trapped less solvent. The findings here are in line with our concept about the formation of pores in each composite as shown in Figure 3.

**Table 2:** Carbon percentage of TiO_2_ anatase standard (Sigma Aldrich, 637254), TiO_2_–5CB composites based on the TGA and experimental data.

**Sample**	**TGA (%)**	**Experimental (%)^a^**
TiO_2_ anatase standard (Sigma Aldrich, 637254)	0.2	-
TiO_2_–5CB synthesized under magnetic field	13.0	12.3
TiO_2_–5CB synthesized without magnetic field	12.8	12.3

a *The amount of 5CB that was added during the synthesis of TiO_2_–5CB*.

**Figure 5:**
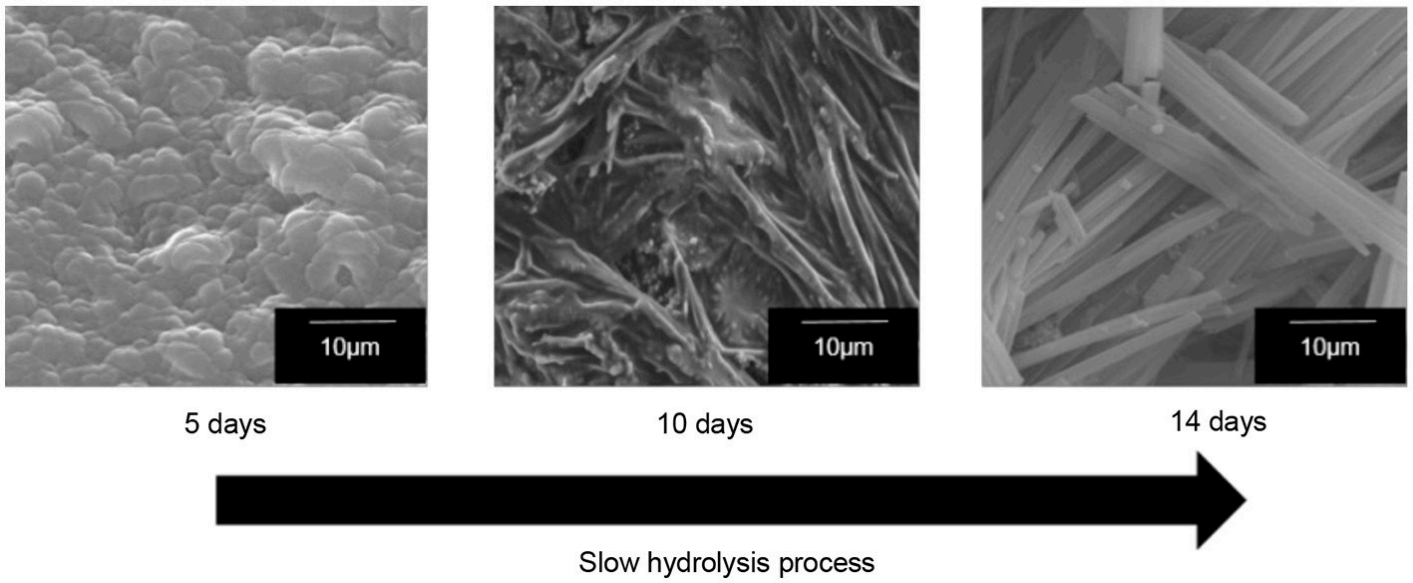
The SEM images of well-aligned 1-D-like TiO_2_–5CB synthesized under magnetic field in slow hydrolysis process.

**Figure 6:**
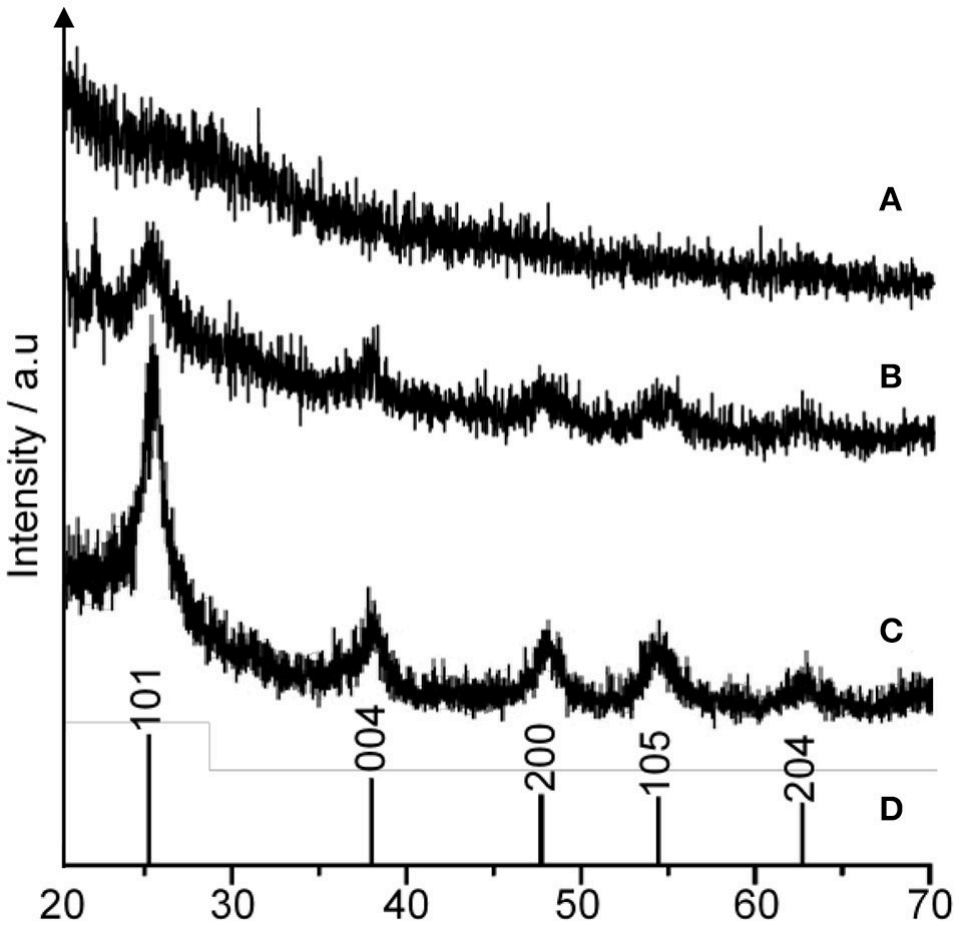
The XRD pattern of TiO_2_–5CB synthesized **(A)** without magnetic field, **(B)** under magnetic field, and **(C)** TiO_2_ anatase standard (Sigma Aldrich, 637254), **(D)** JCPDS card number 21-1272.

The formation of 1-D-like TiO_2_ has a strong relationship with the existence of liquid crystal in a magnetic field. Nevertheless, the condition during the hydrolysis process is very crucial for the formation of 1-D-like TiO_2_ too. It was found that a slow hydrolysis rate is the key factor for the formation of 1-D-like TiO_2_–5CB under a magnetic field. Figure 5 shows the formation of 1-D-like TiO_2_–5CB synthesized under a magnetic field by a slow hydrolysis process. During the slow hydrolysis, it was expected that the orientation of TBOT was templated by the anisotropic medium of 5CB; hence, the 1-D-like TiO_2_ was obtained. Interestingly, the impact of the anisotropic 5CB on the orientation of TiO_2_ molecules can be proven by the XRD study, where an anatase phase was observed in the 1-D-like TiO_2_–5CB composite. As shown in Figure 6, TiO_2_–5CB synthesized without a magnetic field was in an amorphous phase only. Whereas, the peaks representative of the anatase phase were observed for the TiO_2_–5CB synthesized with a magnetic field. This composite was not fully amorphous. It is well established that diamagnetic assemblies having magnetic anisotropy will become oriented in a steady magnetic field to achieve the minimum-energy state (Collings and Hird, 1997). Therefore, the structure of organized molecular assemblies of 5CB liquid crystal can be controlled by a magnetic field. When 5CB molecules receive torque in a magnetic field, they will orientate in the direction where its magnetic energy is minimum. This orientated mesophase acts as the template for subsequence crystallization. This phenomenon made it possible to align a TBOT skeleton through the magnetic orientation of a nematic mesophase of the 5CB used as a template. During the hydrolysis process, the TBOT molecules polymerize slowly in the aligned mesophase. Hence, the ordered structure of TBOT is formed by the arrangement of anisotropic molecules, and the resultant crystallinity increases (Yamaguchi and Tanimoto, 2006). Several studies also reported this similar phenomenon, where the magnetic field affects the crystallinity of synthesized materials such as BiFeO_3_/LDPE (Song et al., 2017), Fe_3_O_4_ (Hong et al., 2007), and CaCO_3_ (Tai et al., 2008). Based on the data obtained, we suggest that the crystallization process is dependent on the orientation of TBOT molecules, and this concept is illustrated in Supplementary Data 4.

**Figure 7:**
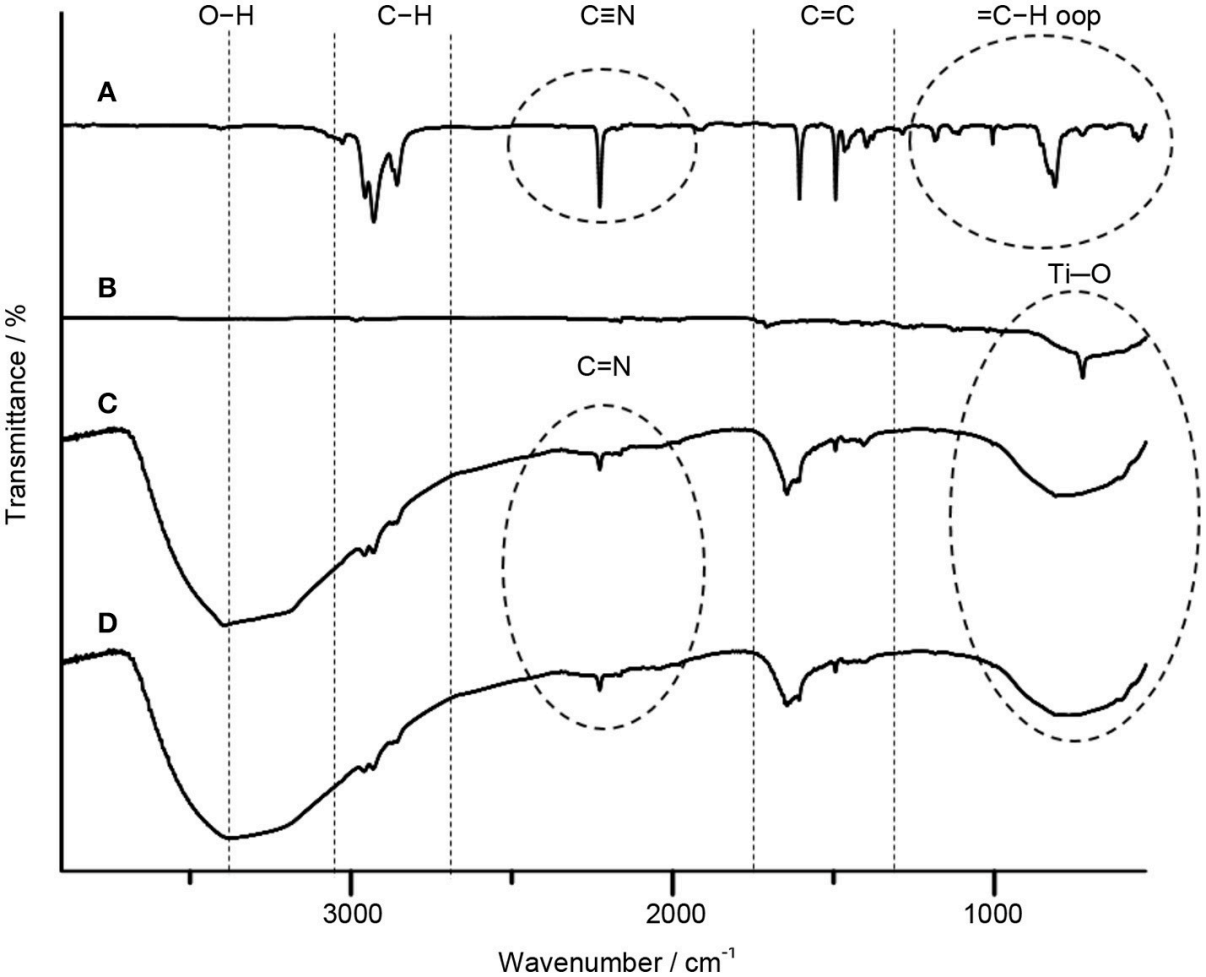
The FTIR spectra of **(A)** 5CB liquid crystal, **(B)** TiO_2_ anatase standard (Sigma Aldrich, 637254), **(C)** TiO_2_–5CB synthesized without magnetic field, and **(D)** TiO_2_–5CB synthesized under magnetic field.

The FTIR spectroscopy is used to further resolve the interfacial interaction in TiO_2_–5CB composites. The FTIR spectra of TiO_2_, 5CB, TiO_2_–5CB synthesized under or without magnetic field are depicted in Figure 7. The broad peaks at around 523–783 cm^−1^ for TiO_2_–5CB synthesized under and without magnetic field corresponded to the Ti–O bond in TiO_2_ (Pavia et al., 2014). Besides that, the FTIR spectra of TiO_2_–5CB synthesized under and without magnetic field also exhibited a broad peak at 3,300–3,400 cm^−1^, which corresponded to the stretching vibration of hydroxyl (O–H) group since the solvent used during the synthesis process was 2-propanol. In addition, the characteristic peaks of 5CB liquid crystal, which were clearly observed, were assigned to as follows: C=N nitriles vibration at 2,226 cm^−1^, = C–H aromatic stretching vibration at 3,026 cm^−1^, C–H stretching vibration at 2,856–2,956 cm^−1^, C = C aromatic stretching vibration at 1,494 cm^−1^ and 1,606 cm^−1^, and C–H aromatic out of plane bend at 553–815 cm^−1^ (Pavia et al., 2014). The peaks of C–H aromatic out of the plane bend at 553–815 cm^−1^ clearly disappeared after TiO_2_ was mixed with the liquid crystals and the peaks of C = N nitriles appeared at 1,645 cm^−1^. This is because the Ti–O bond in TiO_2_ overlapped with this peak since the amount of TiO_2_ precursor used in the synthesis is higher than that of liquid crystals and also due to the occurrence of the interaction between TiO_2_ and liquid crystals. At the same time, the characteristic peaks of 5CB liquid crystal, which are C = N nitriles, C–H stretching vibration, and C = C aromatic stretching, and also Ti–O bond, exhibited a shifting when a comparison was done between the TiO_2_–5CB synthesized under and without magnetic field. The peaks shifted marginally to a higher wave number, suggesting the occurrence of a strong interaction between TiO_2_ and the 5CB liquid crystal when a magnetic field is applied.

**Figure 8:**
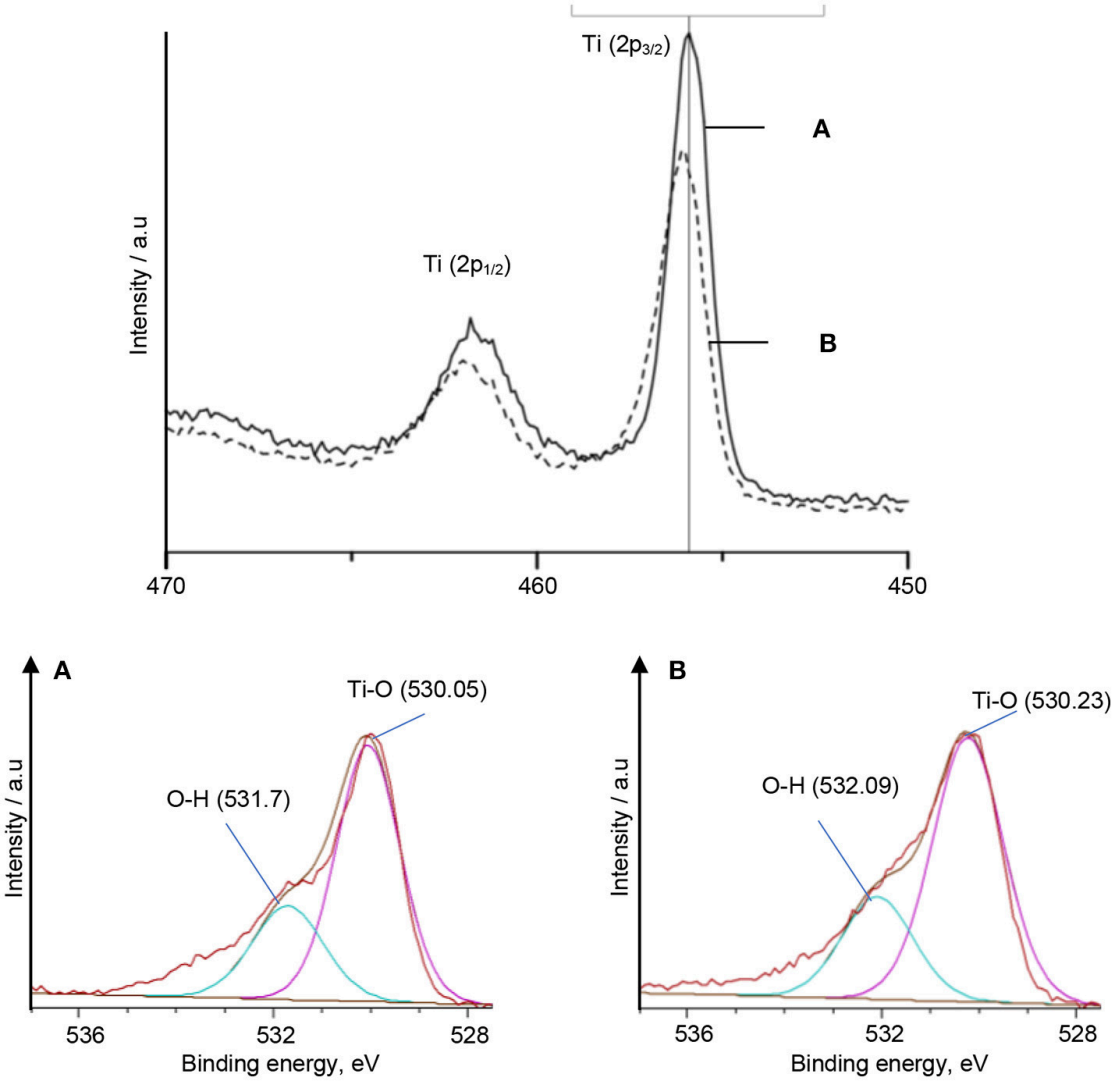
The XPS spectra of Ti and O species in TiO_2_–5CB synthesized **(A)** without magnetic field and **(B)** under magnetic field.

Characterization using XPS was also carried out to strengthen the interfacial interaction discussion on TiO_2_–5CB synthesized under and without magnetic field since the Ti–O bond shifted marginally to a higher wave number in the FTIR spectrum. The XPS analysis was done to validate the interaction that occurs in the Ti–O bond. The high-resolution spectra for Ti 2p and O 1s of TiO_2_–5CB synthesized under and without magnetic field are shown in Figure 8. The binding energy of XPS is calibrated for interpretation. The peak at 456 eV represents Ti 2p^3/2^ and the peak at 462 eV represents Ti 2p^1/2^, while the peak at 530 eV represents O 1s (Moulder et al., 1992). The Ti and O peaks of TiO_2_–5CB synthesized under magnetic field shifted 1.0 eV to a higher binding energy compared with that of TiO_2_–5CB synthesized without magnetic field. This validated the FTIR results that there is the occurrence of a strong interaction between TiO_2_ and 5CB.

**Figure 9:**
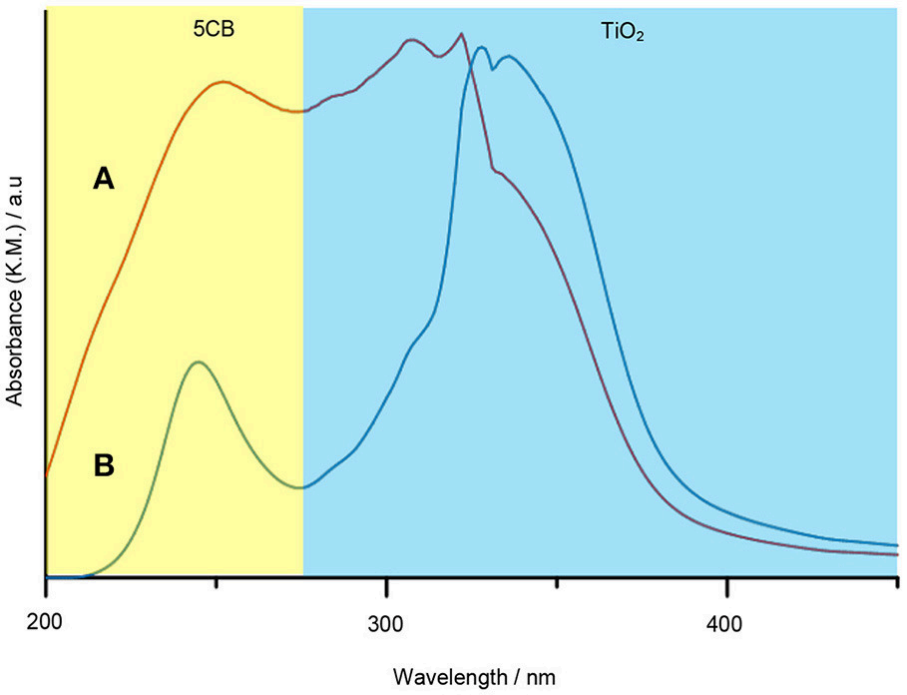
The DR UV–Vis spectra of TiO_2_–5CB synthesized **(A)** without magnetic field and **(B)** under magnetic field.

**Figure 10:**
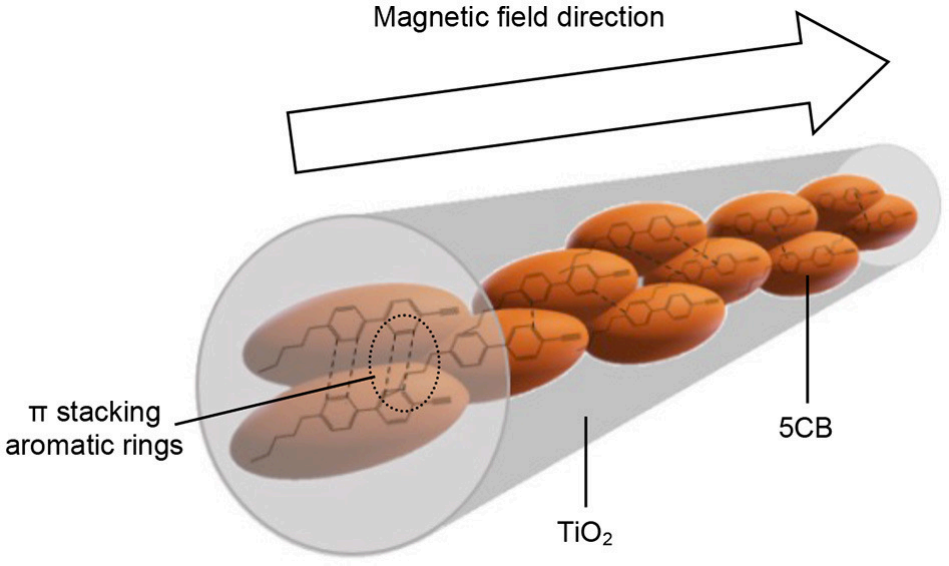
Plausible π stacking aromatic rings for well-aligned 1-D-like TiO_2_–5CB synthesized under magnetic field.

The interaction of 5CB liquid crystal in the TiO_2_ composite samples was further confirmed using DR UV–Vis spectrophotometer. One of the possible interactions that can be evaluated by DR UV–Vis spectroscopy is the chromophore–chromophore interaction and the ð^*^ stacking of the aromatic rings. Figure 9 shows the DR UV–Vis spectra of TiO_2_ synthesized under and without magnetic field. Both spectra show a similar pattern. There are two peaks appearing in the DR UV–Vis spectra, i.e., the peak at 210 to 230 nm and 300 to 330 nm, which belong to 5CB and TiO_2_, respectively. A reduction in intensity for the peak of 5CB at 210 to 230 nm can clearly be seen for the spectrum of TiO_2_–5CB synthesized under magnetic field. This is caused by the quenching phenomenon that occurred in the sample. The 5CB liquid crystal has aromatic rings, which is a stack of aromatic chromophores that experience reduced relative intensity. Thus, when the aromatic rings of 5CB liquid crystal are stacked, interactions between those chromophores occur, causing a reduction in the intensity, known as ð^*^ stacking of the chromophores (Bauer et al., 1976). The chromophores may interact with one another and perturb their electronic transitions. The electronic transition is sensitive to the interactions between two chromophores that approach one another. These interactions can show up in electronic transitions as shifts in wavelength as well as in intensity changes (Bauer et al., 1976). As shown in the SEM image of the TiO_2_ synthesized under magnetic field (see Figure 1B), the TiO_2_ is well-aligned and closely stacked together. This arrangement allows the interaction between the chromophores, which is proven by the DR UV–Vis spectrum of the sample. But, for the case of TiO_2_–5CB synthesized without a magnetic field, the random arrangements did not allow interactions between the chromophores; thus, no quenching can be seen in its DR UV–Vis spectrum. This shows that the alignment of the TiO_2_ influences the interaction of its chromophores. As a result, the plausible chromophore–chromophore interaction of 5CB liquid crystal, which was embedded inside TiO_2_, was illustrated in Figure 10.

### Photocatalytic activity

The photocatalytic activity of TiO_2_ synthesized with liquid crystal as the structure-aligning agent under magnetic field (0.3 T) was evaluated through the photocatalytic oxidation of styrene in the presence of the hydrogen peroxide (H_2_O_2_) as an oxidizing source. The stability of composites under irradiation of UV light was checked for prior to the experiment. The composites were irradiated under UV irradiation produced by a UV lamp (Vilber Lourmat, VL-215-C) with radiation intensity of 2 × 15 W and wavelength of 254 nm for 12 h. The morphology of 1-D-like TiO_2_ composites remained intact after the photoirradiation process. This is due to the photostability properties of the 5CB liquid crystal (Wen et al., 2005) wherein it cannot be photodegraded easily under UV irradiation. The FTIR spectrum for TiO_2_–5CB synthesized under magnetic field after the photoirradiation process shows the functional group of nitriles, -C=N, and aromatic rings, C = C, similar to those detected in the spectrum of TiO_2_–5CB synthesized under magnetic field prior to photoirradiation. It is proven that the same functional groups were retained even after the photoirradiation process although the intensities of the peaks are slightly reduced. This indicates that the structure of the 1-D-like TiO_2_–5CB synthesized under a magnetic field is quite stable and could not be destroyed easily even after the photoirradiation process. It suggests that the 1-D-like TiO_2_–5CB synthesized under magnetic field could be suitable as a photocatalyst.

**Figure 11:**
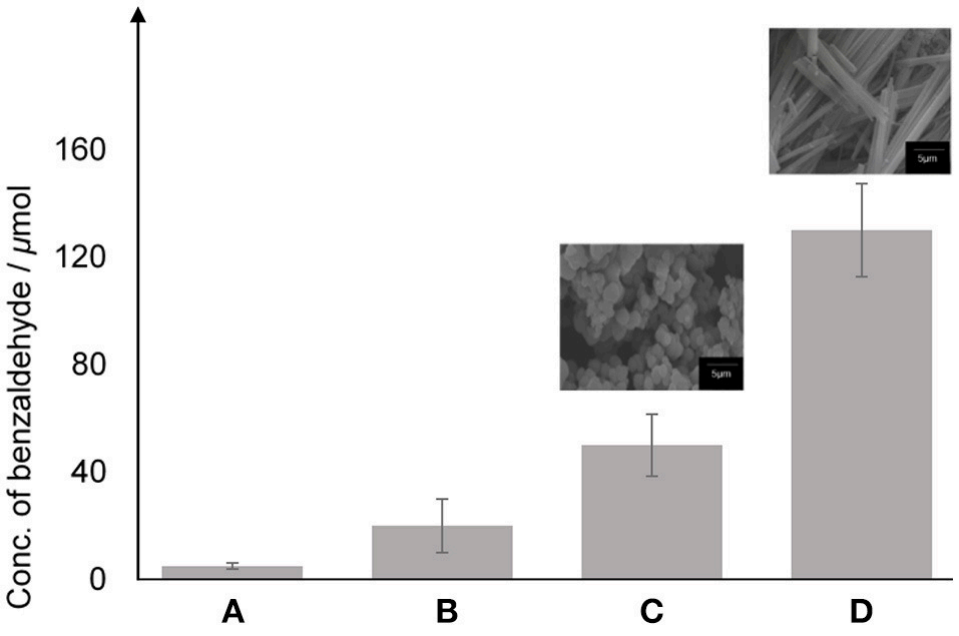
The concentration of benzaldehyde obtained from the photocatalytic oxidation of styrene using photocatalyst **(A)** blank, **(B)** TiO_2_ anatase standard, **(C)** TiO_2_–5CB synthesized without magnetic field and **(D)** TiO_2_–5CB synthesized under magnetic field.

In order to evaluate the photocatalytic performance, three different photocatalysts were chosen; (B) TiO_2_ anatase standard (Sigma Aldrich, 637254), conducted as the reference for this experiment, (C) TiO_2_–5CB synthesized under magnetic field, and (D) TiO_2_–5CB synthesized without magnetic field. A blank sample (A) consisted of only hydrogen peroxide, styrene, and acetonitrile was used as control experiment. Figure 11 shows the conversion of styrene to benzaldehyde in the photocatalytic reaction. The highest concentration (130 μmol) was obtained for the TiO_2_–5CB composite synthesized under magnetic field (0.3 T). This is strong evidence that the 1-D-like structure can improve the photocatalytic power of TiO_2_. The possible reason is that the electronic properties of TiO_2_–5CB have been modified. For another reason, the 1-D-like structure has a limited diffusion direction throughout the structure, which resulted in the unidirectional traveling path of the electron (Thakur et al., 2017). As a result, the recombination of electron and hole is inhibited. Other than that, it can be assumed that the TiO_2_–5CB synthesized under a magnetic field has better interfacial interaction compared with the TiO_2_–5CB synthesized without magnetic field. This good interfacial interaction allows electron transfer between the 5CB liquid crystal and the TiO_2_ during photocatalytic oxidation of styrene, which can enhance the electron transport and delay the recombination of the electron with the hole.

**Figure 12:**
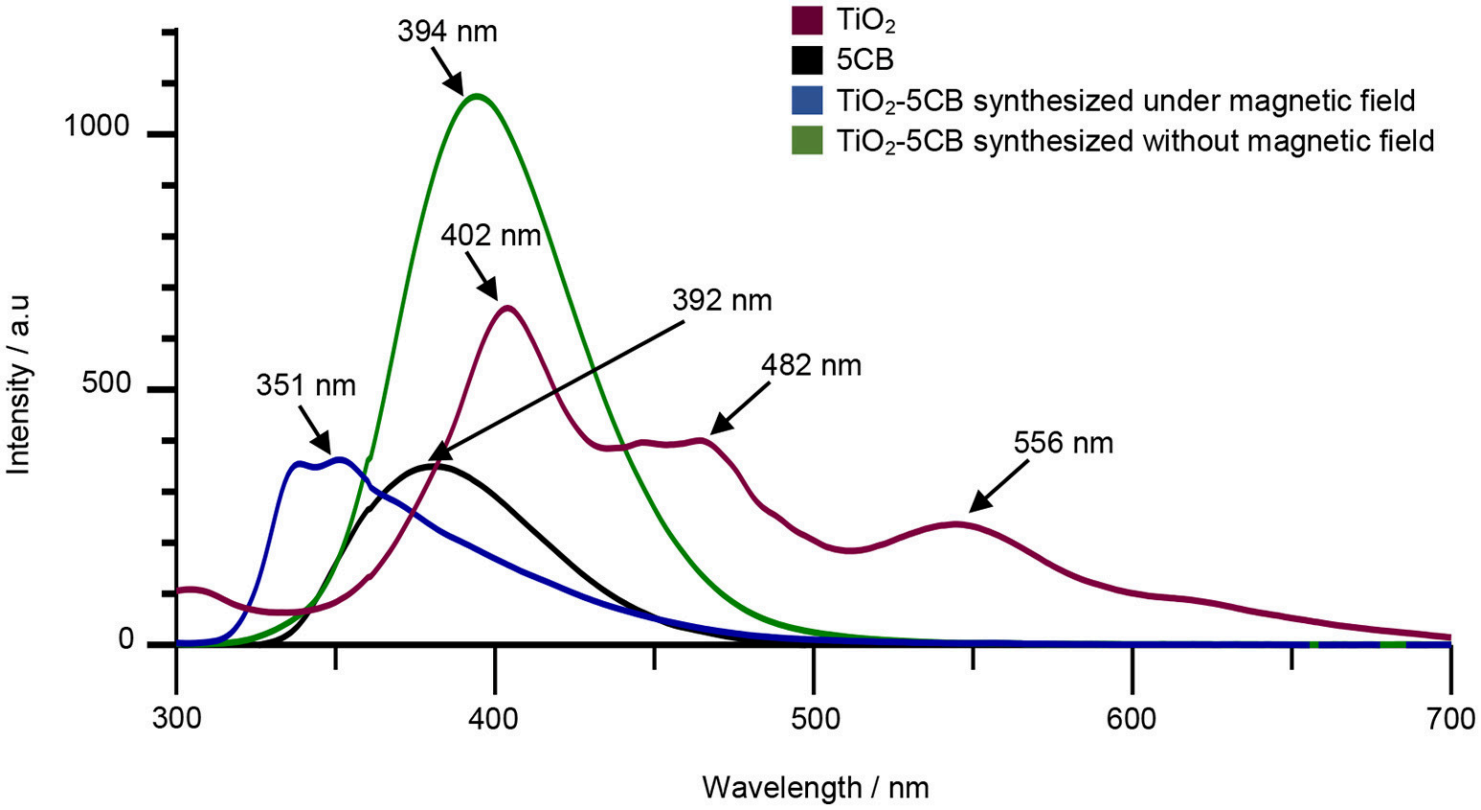
The PL spectra of TiO_2_, 5CB, and TiO_2_–5CB synthesized under magnetic field and TiO_2_–5CB synthesized without magnetic field.

In order to prove our statement, the PL studies were carried out to investigate the efficiency of the charge carrier trapping, migration, and transfer and detecting the behavior of the electron–hole (e^−^/h^+^) pairs in the semiconductor particles. Figure 12 shows the PL spectra of TiO_2_–5CB synthesized under and without a magnetic field, and the bulk TiO_2_ and 5CB as references. The TiO_2_ exhibited three emission peaks, which were at 402, 482, and 556 nm (Cong et al., 2007). The emission peaks at 392 and 363 nm are represented as 5CB (Bezrodna et al., 2017). The emission peak of TiO_2_–5CB synthesized without magnetic field occurred at 394 nm, which was located between the emission peaks of 5CB and TiO_2_. Since the PL emission peak is the result of the recombination of electron–hole, it was indicated that the TiO_2_–5CB synthesized without a magnetic field has a higher recombination rate due to the increasing intensity of the emission peak. Meanwhile, the emission peak of TiO_2_–5CB synthesized under a magnetic field occurred at 351 nm, and it was shifted to a lower wavelength with decreased intensity. It was shown that the decreased intensity of the emission peak leads to the efficient quenching of PL, which can be attributed to the excited electron transfer from the valence band to new levels that exist upper to the conduction band (Cong et al., 2007). For the shifted wavelengths of TiO_2_–5CB synthesized under a magnetic field, this might have been caused by the presence of interactions between TiO_2_ and π-π stacked 5CB (Bezrodna et al., 2017). Thus, it was concluded that electron charge transfer occurred in the well-aligned 1-D-like TiO_2_–5CB synthesized under a magnetic field during UV irradiation in PL analysis.

**Figure 13:**
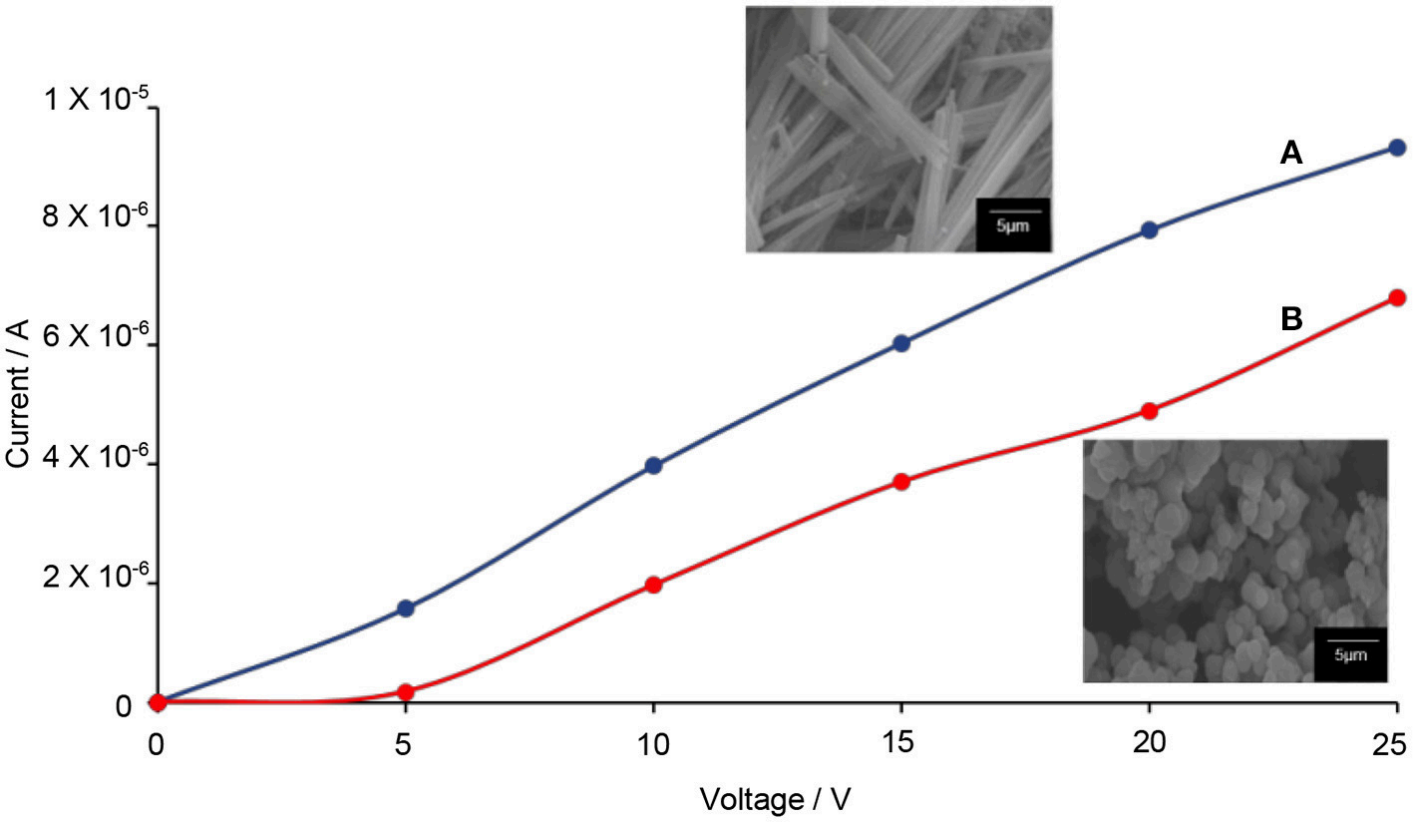
The values of current versus the applied voltage of TiO_2_–5CB synthesized **(A)** under magnetic field and **(B)** without magnetic field.

**Figure 14:**
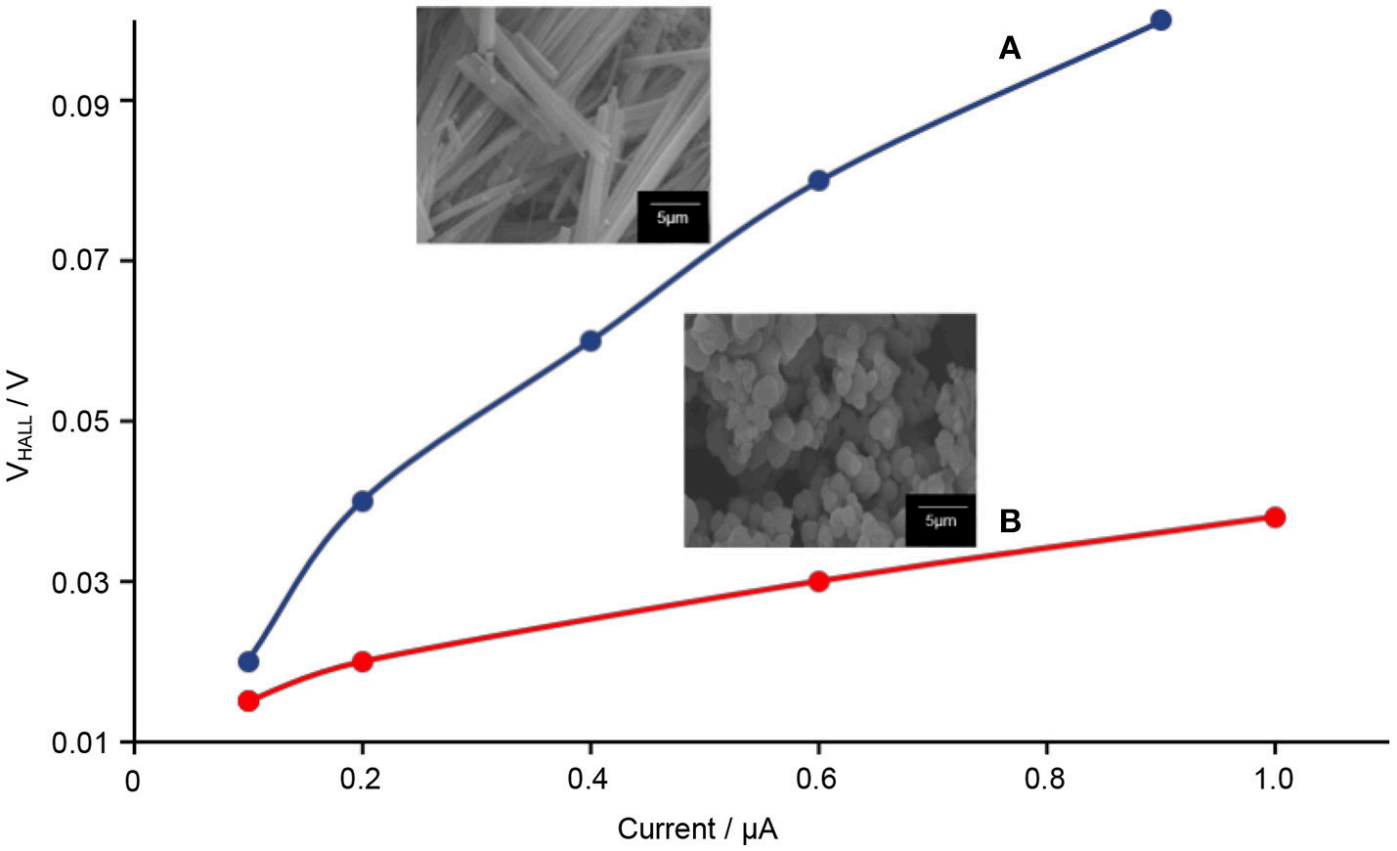
The Hall voltage vs. current of TiO_2_–5CB synthesized **(A)** under magnetic field and **(B)** without magnetic field.

The Hall effect studies can provide useful information on the motional behavior of the electron–hole pairs. From here, the correlation between the 1-D-like structure with the electron–hole pairs can be determined. Prior to the testing, the DC electrical conductivity of TiO_2_–5CB composites was first determined. Figure 13 shows that when a higher voltage was applied to the TiO_2_ composites, the amount of current generated was also higher. These results proved that the TiO_2_ composites allow the flow of electricity. It can be seen that the plotted graph of TiO_2_–5CB, synthesized under magnetic field, shows the highest current due to the difference in shape. The TiO_2_–5CB synthesized under a magnetic field is well-aligned, while the TiO_2_–5CB synthesized without a magnetic field was in an irregular aggregated shape. Thus, it can be concluded that, as the particles of TiO_2_–5CB are arranged neatly next to each other, the flow of electricity has been enhanced and the diffusion of electrons is expected to be improved as well (Liu et al., 2015). Figure 14 shows that the TiO_2_–5CB synthesized under a magnetic field displayed a higher Hall voltage when the current was higher. Once again, this is most probably caused by the arrangement of the TiO_2_–5CB synthesized under a magnetic field, where it is neatly arranged next to each other, because with this arrangement the current can flow smoothly, and, therefore, the Hall voltage becomes higher (Ivanov and Nikolov, 2016). Our results show that the motional behavior of the electron in TiO_2_–5CB of a different shape is quite distinct. For the TiO_2_–5CB composite with 1-D-like structure, the separation of the electron from the hole is expected to be improved due to the better mobility of electron in the unidirectional pathway.

## Conclusions

A model photocatalyst, the 1-D-like TiO_2_ composite, was successfully synthesized through slow hydrolysis under a magnetic field, using a 5CB liquid crystal as the structure-aligning agent. Based on the PL and electrical analyses, it is evident that the 1-D-like TiO_2_–5CB synthesized under magnetic field possesses a slow electron–hole recombination rate. As a conclusion, in this research, the influence of the shape of the TiO_2_ photocatalyst on the photocatalytic properties has been successfully demonstrated. Although the correlation between the 1-D shape of TiO_2_ and the photocatalytic activity has been clearly demonstrated in this research, further work needs to be carried out to obtain 5CB-free 1-D-like crystalline TiO_2_.

## Author contributions

NIA and HN designed the study. NIA developed all the studies under the supervision of HN, SC, NA, and WL. NIA and HN wrote the manuscript with the input from all other authors. HN approved the manuscript.

### Conflict of interest statement

The authors declare that the research was conducted in the absence of any commercial or financial relationships that could be construed as a potential conflict of interest.
